# Strategies for Targeted Delivery of Exosomes to the Brain: Advantages and Challenges

**DOI:** 10.3390/pharmaceutics14030672

**Published:** 2022-03-18

**Authors:** Hojun Choi, Kyungsun Choi, Dae-Hwan Kim, Byung-Koo Oh, Hwayoung Yim, Soojin Jo, Chulhee Choi

**Affiliations:** 1 ILIAS Biologics Inc., Daejeon 34014, Korea; hchoi@iliasbio.com (H.C.); kchoi@iliasbio.com (K.C.); dkim@iliasbio.com (D.-H.K.); bkoh@iliasbio.com (B.-K.O.); hyim@iliasbio.com (H.Y.); sjo@iliasbio.com (S.J.); 2 Department of Bio and Brain Engineering, Korea Advanced Institute of Science and Technology (KAIST), Daejeon 34141, Korea

**Keywords:** exosome, brain delivery, BBB crossing, transcytosis

## Abstract

Delivering therapeutics to the central nervous system (CNS) is difficult because of the blood–brain barrier (BBB). Therapeutic delivery across the tight junctions of the BBB can be achieved through various endogenous transportation mechanisms. Receptor-mediated transcytosis (RMT) is one of the most widely investigated and used methods. Drugs can hijack RMT by expressing specific ligands that bind to receptors mediating transcytosis, such as the transferrin receptor (TfR), low-density lipoprotein receptor (LDLR), and insulin receptor (INSR). Cell-penetrating peptides and viral components originating from neurotropic viruses can also be utilized for the efficient BBB crossing of therapeutics. Exosomes, or small extracellular vesicles, have gained attention as natural nanoparticles for treating CNS diseases, owing to their potential for natural BBB crossing and broad surface engineering capability. RMT-mediated transport of exosomes expressing ligands such as LDLR-targeting apolipoprotein B has shown promising results. Although surface-modified exosomes possessing brain targetability have shown enhanced CNS delivery in preclinical studies, the successful development of clinically approved exosome therapeutics for CNS diseases requires the establishment of quantitative and qualitative methods for monitoring exosomal delivery to the brain parenchyma in vivo as well as elucidation of the mechanisms underlying the BBB crossing of surface-modified exosomes.

## 1. Introduction

The central nervous system (CNS) is one of the most in-demand areas for the development of new therapeutics owing to the increasing occurrence rate of neurodegenerative disorders. However, it remains the most difficult area for drug development because of the blood–brain barrier (BBB), which prevents most of the currently developed drugs from entering the brain parenchyma. The BBB functions as a tight barrier to protect the CNS from potential neurotoxic substances, and regulates the selective transport of specific molecules and nutrients to maintain CNS homeostasis. Water molecules and small ions cross brain capillaries through channels, and small molecules under 500 Da can cross the BBB via passive diffusion [1]. However, macromolecules require specific receptors or transport proteins to facilitate receptor- or adsorptive-mediated transport for entry into the brain parenchyma. The increasing need for new therapeutics for CNS diseases has prompted the investigation of various endogenous transportation mechanisms that can deliver macromolecules across the BBB. The development of novel therapeutics utilizing these transportation pathways has been actively validated in numerous preclinical and clinical studies.

Among the novel therapeutics, exosomes have recently gained attention because of their role as therapeutic vehicles for delivering various active pharmaceutical ingredients to the brain. Exosomes, or small extracellular vesicles (EVs), are a subtype of EVs defined as single-membrane lipid bilayer vesicles generated by vesicle budding into endosomes that mature into multivesicular bodies or by direct vesicle budding from the plasma membrane [2]. Different subtypes of EVs have been identified based on their size and density, which allows separation by methods such as tangential flow filtration, size exclusion chromatography, and differential centrifugation [3]. Nevertheless, careful interpretation is necessary when analyzing different groups of EVs because most EV purification methods cannot determine EVs based on their biogenesis pathways, but rather isolate subtypes of EVs based on their physical properties. Among EVs, exosomes are natural nanoparticles with low immunogenicity that can deliver diverse biological molecules, such as nucleic acids, proteins, lipids, and carbohydrates to target cells [4]. Compared with cell therapy, exosomes possess similar therapeutic efficacy with improved safety profiles in various diseases, such as cancer and ischemia [5,6,7,8,9,10]. To induce targeted delivery to the brain, therapeutic exosomes can be engineered to express various targeting moieties via direct modification methods, such as chemical modification of exosomal surfaces, or indirect modification methods via genetic engineering of exosome-producing cells. The aim of this review is to briefly discuss current engineering strategies for delivering therapeutics across the BBB and highlight recent advances in the targeted delivery of exosomes to the brain.

## 2. Current Strategies for Delivering Therapeutics across the BBB

**Figure 1:**
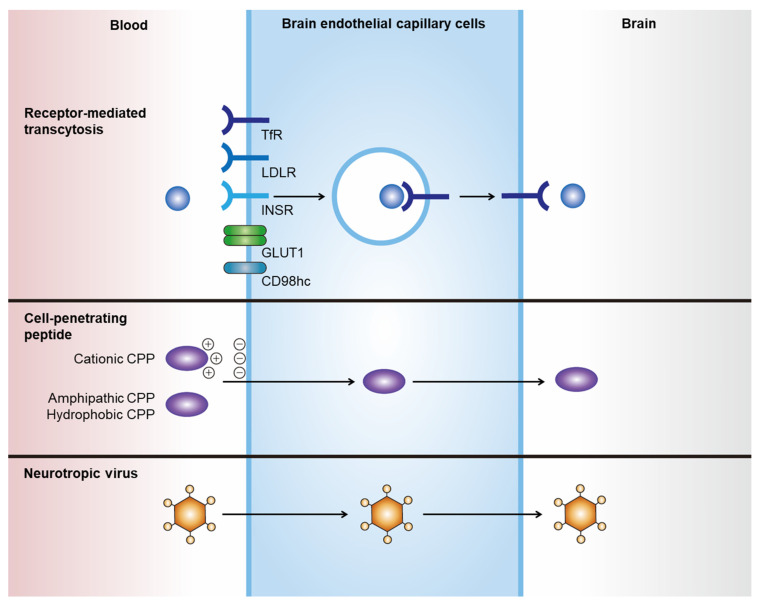
Strategies for delivering therapeutics across the BBB. Noninvasive delivery of therapeutics across the BBB can be achieved by hijacking endogenous transport pathways. RMT-mediated brain delivery of therapeutics can be achieved by expressing specific ligands that bind to receptors and induce transcytosis, such as TfR, LDLR, INSR, GLUT1, and CD98hc. CPPs are a family of various short peptides (fewer than 30 amino acids) that can induce the translocation of macromolecules across cell membranes without interactions with specific receptors. Neurotropic viruses can cross the BBB and invade the brain parenchyma using specific viral components, such as rabies virus glycoprotein.

Noninvasive delivery of therapeutics to the CNS can be achieved by hijacking endogenous transport pathways, such as receptor-mediated transcytosis (RMT) and adsorptive-mediated transcytosis (Figure 1, Table 1) [11,12]. Among these, RMT has been the most investigated and applied route for the transportation of drugs through endothelial cells of the BBB [13]. Various therapeutics, including chemicals, antibodies, polymeric nanoparticles, and exosomes, can incorporate these strategies. Their efficacy in brain delivery has been actively tested in numerous preclinical studies and clinical trials [11].

### 2.1. Receptor-Mediated Transcytosis

Transcytosis is the vesicular crossing of macromolecules from one side of the cell membrane to another [14]. RMT is mediated by the binding of a ligand to a specific receptor, which subsequently induces receptor-mediated endocytosis and further transports invaginated endosomal compartments to the other side of the membrane. Drugs can hijack RMT by expressing specific ligands that bind to receptors that mediate transcytosis. The optimal receptors to be utilized for RMT-mediated BBB crossing are highly and locally expressed on the membrane of brain capillary endothelial cells (BCECs), with low expression on peripheral endothelial cells. However, to date, no ideal receptor has been identified. Nevertheless, highly and ubiquitously expressed receptors on BCECs have shown promising results in RMT-mediated brain delivery in preclinical studies and several clinical trials.

#### 2.1.1. Transferrin Receptor

The transferrin receptor (TfR) is a widely used and validated receptor for the RMT-mediated BBB crossing of therapeutics. Transferrin is an iron-binding glycoprotein that delivers iron to cells by binding TfR. Although TfR is a ubiquitously expressed receptor, proteomics analysis confirmed that TfR is one of the highest expressed receptors that induces transcytosis in mice [17] and human BCECs [18]. The valency and binding sites of TfR-binding moieties should be carefully considered when developing a TfR-mediated brain delivery system. Recent studies have reported that TfR-targeting antibodies with high valency paradoxically have lower BBB crossing efficacy than low-valency antibodies owing to the lysosomal degradation of antibody-bound TfR [19,20]. Degradation of antibody-bound membrane proteins can also occur in other receptors for RMT-mediated CNS delivery, which warrants further investigation. In addition, it is ideal for TfR-targeting moieties to bind to regions that do not interrupt the endogenous binding of TfR to the receptor. TfR-mediated brain delivery has been applied to various therapeutics, such as liposomes [21,22,23] and chitosan nanospheres [24]. In addition, several clinical trials of TfR-mediated brain delivery of therapeutics have shown promising results. For example, clinical trials of the lysosomal enzyme iduronate 2-sulfatase conjugated with anti-human TfR antibody have shown positive results in Hunter syndrome (NCT0312893, NCT03568175, and NCT04251026).

#### 2.1.2. Low-Density Lipoprotein Receptor

The low-density lipoprotein receptor (LDLR) family is mainly responsible for the endocytosis of low-density lipoproteins (LDLs), such as apolipoprotein B (ApoB) and apolipoprotein E (ApoE). Each LDL particle contains a single apolipoprotein surrounded by fat molecules, such as cholesterol, phospholipids, and triglycerides, which mediate the delivery of these fatty acids into cells in need. LDLR is not only a ubiquitously expressed receptor, but is also widely expressed in the brain, rendering it an efficient transporter of therapeutics. The conjugation of ApoB- and ApoE-derived peptides to proteins, such as lysosomal enzymes, has been demonstrated to successfully transport proteins across the BBB [25,26,27,28]. Nanoparticles, such as liposomes, high-density lipoprotein nanocarriers, and polymersomes, functionalized with ApoE-derived peptides, have also shown enhanced BBB crossing through LDLR- and LDLR-related protein 1 (LRP1)-mediated transcytosis in BCECs [29,30,31,32,33,34,35]. ApoB-derived peptides have also exhibited targeted delivery of siRNA and nanoparticles to the brain parenchyma [36,37]. Angiopep-2 is a 19-amino acid peptide originating from the Kunitz domain of bovine protein aprotinin, which binds to LRP1. LRP1 is widely expressed in human and mouse BCECs [18] and gliomas [38], making angiopep-2 an attractive targeting moiety for various nanoparticles for brain delivery and glioma targeting [39,40,41,42,43,44,45,46,47].

#### 2.1.3. Insulin Receptor

Insulin receptor (INSR) is widely expressed in various tissues. A recent study comparing the expression levels of various receptors mediating transcytosis showed that only INSR was overexpressed in human brain microvessels compared to brain parenchymal and peripheral tissues [48]. A similar pattern was observed in mice, in which INSR, insulin-like growth factor-1 receptor, and LRP8 were highly expressed in brain microvessels compared to those in peripheral tissues [48]. The development of a humanized INSR antibody (HIRMAb), which showed effective delivery in the primate brain after intravenous injection, has accelerated the use of INSR for the brain delivery of drugs [49]. The lysosomal enzyme α-L-iduronidase (IDUA), which is dysfunctional in patients with mucopolysaccharidosis type I, was conjugated to HIRMAb; HIRMAb-conjugated IDUA delivered 1.2% of the injected dose to the brains of rhesus monkeys, whereas IDUA alone resulted in no delivery into the brains [50]. HIRMAb-conjugated IDUA also showed plasma pharmacokinetic profiles comparable to those of human IDUA (laronidase) [51], and an open-label phase 1–2 trial demonstrated a clinical evidence of cognitive and somatic stabilization in patients with mucopolysaccharidosis type I after 52 weeks of intravenous treatment, although few adverse effects, such as infusion-related reactions and transient hypoglycemia, occurred [52].

#### 2.1.4. Other Membrane Proteins

CD98 heavy chain (CD98hc), also known as 4F2 antigen, is a heterodimer membrane protein consisting of a type 2-glycosylated 80-kDa heavy chain linked to a 37-kDa light chain by disulfide bonds [53,54]. CD98hc is highly expressed in human BCECs compared to TfR1, INSR, and LRP1 [18]. CD98hc is expressed on both the apical and basolateral membranes, and can bind and transport amino acids containing CD98 light chains across the BBB [55,56]. A recent study revealed via proteomic analysis that CD98hc is highly expressed in mouse BCECs and that systemic administration of bispecific antibodies targeting CD98hc and β-secretase 1 leads to efficient brain delivery and brain amyloid-beta reduction [17]. This study also showed that targeting CD98hc is more efficient in brain delivery than targeting TfR [17]. CD98hc binding did not alter the endogenous expression and function of CD98hc [17], whereas previous reports have shown that TfR antibodies induce the lysosomal degradation of TfR in an affinity-dependent manner [20].

Glucose transporter 1 (GLUT1), also known as SLC2A1, is a glucose transporter highly expressed on both the apical and basolateral membranes of BCECs [57]. The human brain depends almost entirely on glucose as an energy source, consuming approximately 20% of the total glucose in the body [58], which requires high expression of GLUT1 on the BCECs for efficient glucose transport. Glucose derivatives have been utilized for the BBB crossing of various nanoparticles, such as liposomes [59,60,61,62,63] and micelles [64,65,66], which implies that GLUT1 could be an attractive target receptor for the CNS delivery of therapeutics.

### 2.2. Cell-Penetrating Peptides

Cell-penetrating peptides (CPPs), or protein transduction domains, are a family of short peptides (<30 amino acids) that can induce the translocation of biologically active macromolecules across cell membranes without interacting with specific receptors [15,16]. Although no consensus has been reached regarding the taxonomy of CPPs, they can generally be categorized into three classes based on their physicochemical properties: cationic, amphipathic, and hydrophobic [15]. The cationic class is mainly composed of peptides with positive charges, such as arginine and lysine, that can interact with negatively charged plasma membranes. The transactivator of transcription (TAT) protein of HIV-1 was the first CPP observed to be internalized into cells in vitro in 1988 [67,68], and it has since been widely investigated as an inducer of intracellular delivery of therapeutics. Amphipathic CPPs are the most commonly found CPPs in nature, and they contain polar and nonpolar amino acid regions [16]. Hydrophobic CPPs contain nonpolar hydrophobic residues that induce cell penetration by interacting with the hydrophobic domains of plasma membranes. The apical surface of cerebral capillaries is densely covered with a negatively charged glycocalyx, which renders positively charged CPPs an efficient transporter of drugs through the BBB [69]. However, several issues must be addressed when using CPPs for brain delivery, such as their low tissue specificity and cellular toxicity. CPP-conjugated drugs show widespread biodistribution owing to their lack of tissue specificity. In addition, the cytotoxicity of CPPs is a major concern [70], as shown in the case of amphipathic CPP model amphipathic peptide, which induces damage to the cellular membrane, resulting in the leakage of cellular components and subsequent cell death [71].

### 2.3. Neurotropic Virus

Neurotropic viruses can cross the BBB and invade the brain parenchyma, which prompted the investigation of viruses or viral components as transporters for the brain delivery of therapeutics. For instance, peptides derived from rabies virus glycoprotein (RVG) exhibit efficient penetration through the BBB and target neurons [72]. Although the exact BBB crossing mechanism is unknown, it is expected to occur via neuronal acetylcholine receptor-mediated RMT [72]. RVG has been utilized for the delivery of various nanoparticles through the BBB, including liposomes [73], layered double hydroxide [74], porous silicon nanoparticles [75], and exosomes [76]. However, its biological safety and efficacy should be investigated in preclinical studies for clinical translation.

## 3. Targeted Delivery of Exosomes to the Brain

### 3.1. Natural Brain Delivery of Exosomes to the Brain

Unmodified exosomes from various cell types show <1% delivery to the brain after systemic injection [77,78], implying that exosomes have a natural tendency to bypass the BBB. This finding was also observed by our group, although the exact mechanism by which naïve exosomes cross the BBB is unknown. Recent studies have shown that exosomes originating from different parental cells have different organ and tissue tropisms [77,79,80,81]. Moreover, the specific membrane proteins or molecules of exosomes responsible for the inclination towards specific organs are not fully known. Nevertheless, altering the cell source of exosomes may be a useful strategy to induce brain delivery. Neural stem cell-derived EVs demonstrated enhanced CNS delivery compared with mesenchymal stem cell-derived EVs in a murine stroke model [82]. Based on these observations, exosomes originating from BCECs or brain tumor cells loaded with doxorubicin were tested for the targeted delivery of doxorubicin to brain tumor in a zebrafish model [83].

Transport across the BBB is enhanced under specific pathological conditions. In mice exhibiting brain inflammation, macrophage-derived exosomes showed over three-fold increased delivery to the brain compared to those in normal mice [84]. Enhanced brain delivery is achieved through the interaction of lymphocyte function-associated antigen 1, intercellular adhesion molecule 1, and C-type lectin receptors expressed on macrophage exosomes with BCECs [84]. In an in vitro transwell assay, unmodified naïve exosomes demonstrated enhanced endocytosis and subsequent crossing through BCECs in a tumor necrosis factor-α-induced stroke-like inflammation model [85]. As unmodified exosomes exhibit potential for brain delivery without additional modifications, their efficacy for BBB crossing should be further validated in preclinical studies.

### 3.2. Brain Delivery of Engineered Exosomes by Receptor-Mediated Transcytosis

**Figure 2:**
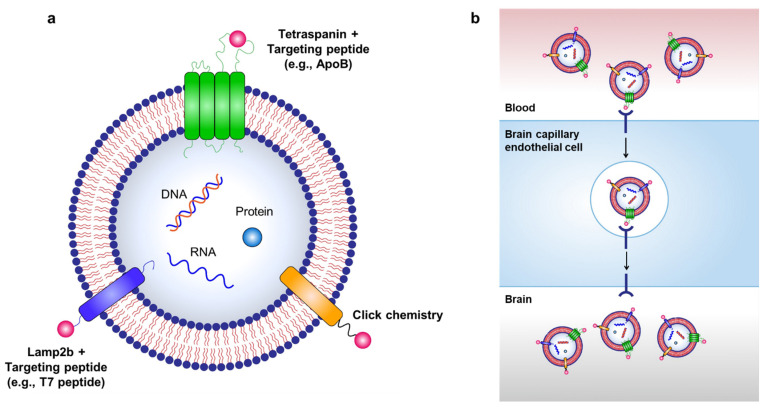
Strategies for targeted delivery of therapeutic exosomes to the brain. (**a**) Targeted delivery of exosomes to the brain can be achieved by labeling various targeting moieties on the surface of exosomes. Therapeutic exosomes can be engineered to express various targeting moieties via chemical modifications, such as click chemistry, or via genetic modification of exosome-producing cells to express targeting peptides fused with exosomal membrane-associated components, such as Lamp2b and tetraspanins. (**b**) RMT can be used to transport exosomes to the brain via labeling of targeting peptides on the surface of exosomes.

**Figure 3:**
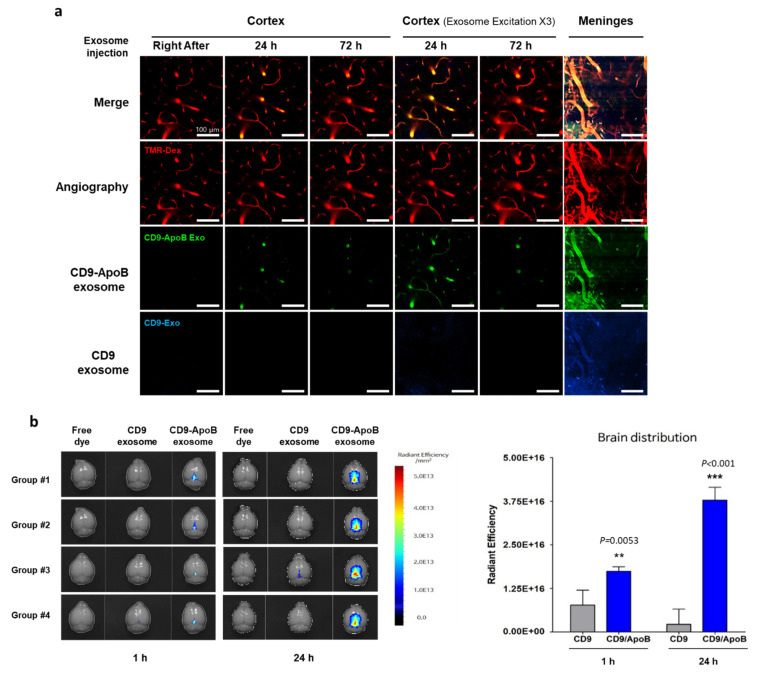
Brain-targeted delivery of exosomes modified with ApoB peptide. (**a**) Exosomes (CD9 or CD9-ApoB) were isolated from transiently transfected Expi293F cells with *CD9* or *CD9/LEL170-ApoB* expression vectors, respectively, and purified using an Amicon Ultra-4 Centrifugal Filter. DiO-labeled CD9 exosomes (blue) and DiD-labeled CD9-ApoB exosomes (green) were intravenously injected (at 1 × 10^10^ particles each) to C57BL/6 mice. The cortical vascular images of mouse brain in vivo through the cranial window were obtained using intravital confocal microscopy (IVIM Technology, Daejeon, Korea). Cerebral angiography was obtained using TMR-dextran (Red). (**b**) Exosomes (CD9 or CD9-ApoB) were stained with DiD, and labeled exosomes (1 × 10^10^ particle number/head) were intravenously injected to C57BL/6 mice. The brain distribution of exosomes was determined via fluorescence imaging by VISQUE^®^ InVivo Smart-LF, an in vivo optical imaging system [91]. Differences between groups were compared using two-way analysis of variance with Bonferroni’s multiple comparison test. Data are expressed as mean ± SEM. ** *p* < 0.01, *** *p* < 0.001.

Targeted delivery of exosomes to the brain can be achieved through various exosome surface modifications (Figure 2). As hijacking RMT is a widely used strategy for delivering therapeutics across the BBB, it can also be used for transporting exosomes to the brain via labeling of targeting peptides on the surface of exosomes. For example, Kim et al. used a T7 peptide for the delivery of exosomes (T7-exo) [86]. T7 peptide is a TfR-binding peptide with the sequence HAIYPRH, which does not disturb the binding of transferrin to TfR [87,88]. By conjugating T7 peptide to Lamp2b, T7-exo demonstrated superior targeting of intracranial glioblastoma in rat models after intravenous injection compared to unmodified exosomes or RVG-labeled exosomes [86]. Recently, our group utilized the LDLR-mediated transcytosis pathway for the delivery of exosomes by generating ApoB-labeled exosomes via conjugation of ApoB with tetraspanin CD9 (unpublished data). Tetraspanins, such as CD9, CD63, CD81, and CD82, are abundant transmembrane proteins expressed on exosomes, and they consist of four membrane-spanning domains and two extracellular loops termed the short extracellular loop (SEL) and large extracellular loop (LEL) [89]. Tetraspanins can be modified to contain targeting peptides by incorporating the peptides into the extracellular loops of tetraspanin [90]. We transfected Expi293F cells with plasmids encoding *CD9* as a control or *CD9/LEL170-ApoB*, in which ApoB was inserted between the 170–171 amino acid of CD9, to generate ApoB-expressing exosomes. Control exosomes (CD9 exosomes) or ApoB-expressing exosomes (CD9-ApoB exosomes) were isolated and purified from the supernatants of transfected Expi293F cells. To characterize the tissue distribution of the injected exosomes, we labeled CD9 exosomes with DiO and CD9-ApoB exosomes with DiD lipophilic fluorescent dyes, which were injected intravenously into mice. A laser scanning intravital confocal microscope was used to visualize the labeled exosomes in the cerebral cortex of mice by implanting a cranial window. We observed the accumulation of CD9-ApoB exosomes in the cortical blood vessels compared to CD9 exosomes, which were not detected in the vessels (Figure 3a). Next, we examined the biodistribution of surface-engineered exosomes in the mouse brain. DiD-labeled CD9 or CD9-ApoB exosomes were intravenously injected into mice, and the fluorescence intensity was analyzed using a preclinical optical imaging system. As shown in Figure 3b, the fluorescence intensity CD9-ApoB exosomes in the brain was significantly higher than that of the control CD9 exosomes, indicating prolonged retention in the brain for 24 h. These findings revealed the improved CNS-targeting capability of the surface-modified exosomes that hijacked the RMT pathway. Further studies are needed to determine the delivery efficacy of various other receptors for RMT-mediated brain delivery of exosomes.

### 3.3. Other Strategies for Brain Delivery

Neurotropic virus-derived peptides, such as RVG, have been used to induce brain-targeting of exosomes in several preclinical studies. In one study, brain delivery of siRNA-loaded exosomes was achieved by expressing RVG at the exosomal membrane and fusing it with Lamp2b, an exosomal membrane protein [76]. The exact BBB crossing pathway has not been shown; however, modified exosomes demonstrated efficient delivery of siRNA to neurons, microglia, and oligodendrocytes in mouse brain [76]. In another study, the same group used a similar approach to deliver siRNA for α-synuclein (α-Syn) to the brain of α-syn transgenic mice [92]. Further studies are needed to identify safety issues associated with the use of virus-derived peptides as therapeutic agents.

Peptides that bind to specific membrane proteins can also be used for exosome modification. For example, c(RGDyK) peptide, which binds to integrin α_v_β_3_ that is highly expressed in BCECs under ischemic conditions, was labeled on the surface of mesenchymal stem cell-derived exosomes through click chemistry [93]. Click chemistry, also known as copper-catalyzed azide-alkyne cycloaddition, is an efficient covalent reaction of an alkyne and an azide residue to form a stable triazole linkage, and can be applied to attach various targeting moieties to the surface of exosomes [94,95,96,97,98]. c(RGDyK) peptide-labeled exosomes exhibited 11-fold enhanced delivery to the ischemic region of the brain compared with scrambled peptide-labeled exosomes in a mouse stroke model [93].

## 4. Conclusions

Exosomes are gaining attention because of their potential as next-generation nanoparticles for treating CNS diseases owing to their potential for natural BBB crossing and broad surface-engineering capability. Various technologies to efficiently incorporate drugs and active pharmaceutical ingredients into exosomes are being actively developed [4,99,100]. In addition, various preclinical studies have investigated engineering strategies for targeted delivery of exosomes to specific organs and tissues [101]. Exosomes carry various membrane proteins (e.g., CD9 [102], CD63 [103], PTGFRN [104], and Lamp2b [76]) and lipids (e.g., phosphatidylserine [105]) that can be utilized for the surface engineering of various targeting moieties. Engineered exosomes possessing targetability to the brain have shown promising results for CNS delivery in preclinical studies; however, they also require intense evaluation through well-designed clinical trials. For the successful development of clinically approved exosome therapeutics for CNS diseases, the establishment of imaging methods for quantitative/qualitative monitoring of exosomal delivery to the brain parenchyma in vivo and uncovering the detailed BBB crossing mechanisms of exosomes is needed.

## Figures and Tables

**Table 1:** Current strategies for delivering therapeutics across the BBB.

BBB Crossing Strategies	Summary
Receptor-mediated transcytosis	- Transcytosis is the vesicular crossing of macromolecules from one side of the cell membrane to the other [14]. - Therapeutics can achieve RMT-mediated brain delivery by expressing specific ligands that bind to receptors inducing transcytosis, such as TfR, LDLR, and INSR [11]. - TfR is responsible for intracellular transport of transferrin and is the most used and validated receptor for RMT-mediated BBB crossing of therapeutics. - LDLR is a ubiquitously expressing receptor and widely expressed in the brain. It is also responsible for the endocytosis of LDLs, such as apolipoprotein B and apolipoprotein E. - INSR is also a widely expressed receptor in various tissues and in the brain microvessels.
Cell-penetrating peptides	- CPPs are a family of various short peptides (fewer than 30 amino acids) that can induce the translocation of macromolecules across cell membranes without interactions with specific receptors [15,16]. - Several issues need to be addressed when using CPPs for brain delivery of therapeutics, such as low tissue specificity and cellular toxicity.
Neurotropic virus	- Neurotropic viruses can cross the BBB and invade the brain parenchyma using specific viral components, such as rabies virus glycoprotein. - Biological safety and clinical efficacy of viral components in the brain delivery of therapeutics should be investigated in more preclinical studies.

## Data Availability

The datasets generated and/or analyzed during the current study are available from the corresponding author upon reasonable request.
